# Predictive equation of metastasis in patients with malignant ovarian epithelial tumors with the Ca-125 marker

**DOI:** 10.1186/s12885-018-4499-y

**Published:** 2018-05-24

**Authors:** Juan Fernando Sánchez Vega, Magdali del Rocío Murillo Bacilio, Adrián Santiago Vintimilla Condoy, Araceli Miroslava Palta González, José Alfredo Crespo Astudillo, Franklin Geovany Mora-Bravo

**Affiliations:** 1 Rural Doctor Ministry of Public Health of Ecuador, Zone 6, Cuenca, Ecuador; 2 Department of Pathology of the Institute for the Fight against Cancer Society -SOLCA-Cuenca, Cuenca, Ecuador; 3 School of Medicine, University of Cuenca, Av. 12 de abril and Paraíso Street, 010201 Cuenca, Ecuador; 4 School of Medicine, University of Azuay, Cuenca, Ecuador; 5 Pablo Jaramillo Foundation, Complementary Health Network, Cuenca, Ecuador

**Keywords:** Prediction of metastasis, Ovarian Cancer, Cancer antigen 125

## Abstract

**Background:**

Cancer antigen (CA) 125 (CA-125) is used in ovarian cancer detection and monitoring, whose serum level has a positive correlation with tumor stage. The aim of this study was to obtain a prediction metastasis equation in a group of patients with ovarian cancer based on Ca-125.

**Methods:**

A 2-group comparative observational study was conducted at a single oncologic institution (SOLCA) in Cuenca-Ecuador. All patients who were diagnosed with ovarian cancer between January 1996 and December 2016 were included in the current study. Group 1 (G1) patients with the I and II International Federation of Gynecology and Obstetrics (FIGO) stage and Metastasis Group (MG), with III and IV stage, were subdivided. A logistic regression equation was performed to predict metastasis based on Logarithm of serum Ca-125 levels.

**Results:**

We included 85 cases in G1 and 64 patients in MG, with 47.8 ± 15 years (G1) and 57.5 ± 13.6 years (MG) of age (*P* < 0.001). Mortality in G1 was 2 cases (3.1%) and 53 cases (62.4%) in MG (P < 0.001). The CA-125 serum level was 163.5 ± 236 in G1 and 1220.9 ± 1940 u / ml in MG (P < 0.001). The equation to predict metastasis = (Age*0.053) + [(Logarithm Ca-125 value) * 1.078] − 8.163 with an OR 2.940 (CI 95% 2.046–4.223) *P* < 0.001. The sensitivity of the equation was 82.4% and the specificity was 79.7%.

**Conclusions:**

It is possible to predict the presence of metastasis in a group of patients with ovarian cancer based on Ca-125.

**Electronic supplementary material:**

The online version of this article (10.1186/s12885-018-4499-y) contains supplementary material, which is available to authorized users.

## Background

Ovarian cancer is one of the ten leading causes of death worldwide, this pathology occurs in all ages including childhood and adolescence, most cases are diagnosed in very advanced stages [1]. In the detection and monitoring of ovarian cancer, the tumor marker Ca-125 is used, but because of its low sensitivity (70%) and specificity (90%) [2], it is used in conjunction with other methods such as imaging such as transvaginal doppler ultrasound [3, 4], improving the diagnosis up to 90% of cases of ovarian cancer [4, 5]. On the other hand, it has been used as a prognostic factor for recurrence and survival, as well as an indicator of disease progression [6], there being a relationship between the tumor stage and the increase in the value of Ca-125 [7]. The level of normality of Ca-125 has been standardized in: less than 35 IU / ml in the postmenopause [8] and 65 IU / ml in the premenopause [3]. The majority of primary ovarian neoplasms originate in the Müller epithelium [9]. The classification is based on the differentiation and extension of epithelial proliferation, with three main histological types: Serous, Mucinous and Endometrioid [10]. Depending on the type of ovarian cancer, differences in serum concentration are observed, for example abnormal levels of Ca-125 are observed in 99% of serous carcinoma cases classified from I to IV in the progression of their clinical stage. Patients with clinical stage I serous carcinoma show normal values of Ca-125 in 11% of cases, while in clinical stage IV, all patients with serous subtype show abnormal Ca-125 values. The total proportion of abnormal Ca-125 values (stages I to IV) was 89% in the endometrioid subtype of ovarian carcinoma, while patients with FIGO stage I endometrioid carcinoma showed 19% of Ca levels. -125 normal. As in the serous subtype, endometrioid ovarian carcinoma shows 100% abnormal Ca-125 in clinical stage IV [4]. There is a relationship between the tumor stage and the increase in the value of Ca-125, with 50% of patients in stage I having an increase, 75% in stage II, 90% in stage III and 98% in Stage IV [7]. There is a need to establish an index to improve cancer care in low/middle income countries where there is limited/no availability of cross-sectional imaging - an accurate index would facilitate triage of patients to a facility with the necessary oncological expertise for optimal surgery and/or chemotherapy (including potentially neo-adjuvant chemo). With this background, the present study aims to perform an equation to predict patients with ovarian cancer who have metastasized on the basis of Ca-125 as a predictor variable.

## Methods

The present in an observational study conducted at the Cancer Institute SOLCA in the city of Cuenca - Ecuador, province of Azuay. Clinical histories of patients diagnosed with Ovarian Cancer who were treated in the 1996–2016 period were reviewed. Cases with insufficient data were excluded for the analysis. We collected demographic variables, type of ovarian cancer, stage and serum levels of Ca-125. The classification of the stages was the one adopted by the FIGO [11] described below to form the study groups:

- Group 1: patients with stage I and II.
- Group 2: patients with stage III and IV.
- Stage I: Stage in which the tumor is confined to the ovaries.
- Stage II: Stage in which the tumor involves one or both ovaries, with extension adjacent to pelvic tissues.
- Stage III: The tumor involves one or both ovaries, with cytology or confirmed histology of spreading out of the pelvis and with metastases to the retro peritoneum and / or lymph nodes.
- Stage IV: Advanced stage of cancer in which there is metastasis confirmed at a distance that excludes peritoneal metastasis.

The analysis of post-harvest information was tabulated and processed using the IBM SPSS Statistics license-free software updated to version 15.0. For the analysis of qualitative variables such as sex, residence, histological type, stage, value of Ca-125 and associated factors, a distribution of relative and absolute frequencies was performed. For analysis of the relationship between the value of Ca-125 versus age, histological type, stage and associated factors, we used chi-square and bivariate analysis. CA-125 value was transformed into a logarithm of base “e” for the regression eq. *P* values ​​less than 0.05 were considered statistically significant. Logistic regression was performed between the groups of Stages I and II versus Stages III and IV to predict the presence of the last stages using Ca-125. The project did not imply any risk for the patients since we worked on clinical history only and this report ensures the confidentiality of identity and the management of the database. The Bioethics Committee of the Cancer Institute SOLCA-Cuenca approved the present study as well as obtaining the permission of the director of the Hospital.

## Results

In the study, 149 cases were enrolled, of which 85 patients were classified as having ovarian cancer without metastasis and 64 patients in the metastasis group. The mean age of the group without metastasis was 47.8 ± 15 years and the group without metastasis was 57.5 ± 13.6 years (*P* < 0.001). With a median of 4 pregnancies in group 1 with an interquartile range of 5 and with 4 pregnancies and an interquartile range of 5 in group 2 (*P* = 0.796). There were no differences between marital status and place of residence between the groups (Table 1). Additionally, the family history of cancer was not different between the groups (Table 1). The histological type of ovarian cancer predominant in both groups was serous (Table 2), with a greater group of women in premenopause in group 1 (Table 1). In group 1 there were 52 patients (81.3%) in Stage I and 12 patients (18.8%) in stage II, in group 2 there were 49 patients (57.6%) in stage III and 36 patients (42.4%) in stage IV. Mortality in group 2 was higher than in group 1 (Table 2). The Ca-125 antigen was positive in 42 cases in group 1 (65.6%) and in 79 cases (92.9%) in group 2 (*P* < 0.001), with an average of 163.5 ± 236 u / ml in group 1 and of 1220.9 ± 1940 u / ml in group 2 (*P* < 0.001).

**Table 1:** Demographic data and background of the study groups

	Group 1Stages I & II-FIGO	Group 2 Advanced ovarian cancer group Stages III & IV-FIGO	*P*
	*n* = 85	*n* = 64	
Civil Status			
Single	20 (23.5%)	17 (26.6%)	0.829
Free union	3 (3.5%)	3 (4.7%)	
Married	42 (49.4%)	34 (53.1%)	
Divorcee	4 (4.7%)	2 (3.1%)	
Widow	16 (18.8%)	8 (12.5%)	
Place of residence			
Cuenca	39 (45.9%)	24 (37.5%)	0.399
Machala	9 (10.6%)	4 (6.3%)	
Gualaceo	3 (3.5%)	1 (1.6%)	
Paute	1 (1.2%)	3 (4.7%)	
Azogues	5 (5.9%)	7 (10.9%)	
Others	28 (32.9%)	25 (39.1%)	
Cancers Family Antecedents	20 (23.5%)	18 (28.1%)	0.524
Breast Cancer Family History	4 (4.7%)	6 (9.4%)	0.493
Hormonal Therapy History	3 (3.5%)	3 (4.7%)	0.722
Biological state			
Premenopausal vs Postmenopausal	20 (23.5%)	27 (42.2%)	0.015

**Table 2:** Histological type of Ovary Cancer in the study groups

Variable	Group 1 Stages I & II-FIGO *n* = 85	Group 2 Advanced ovarian cancer group Stages III & IV-FIGO *n* = 64	*P*
Histological type			
Serous	75 (88.2%)	46 (71.9%)	0.024
Mucinous	9 (10.6%)	10 (15.6%)	
Endometroid	0	5 (7.8%)	
Clear cells	1 (1.2%)	1 (1.6%)	
Transitional cells	0	2 (3.1%)	

### Metastasis probability

The metastasis probability (≥0.50) was established as:

$$ P=\frac{1}{1+{\mathit{\exp}}^{-F}} $$

Where F is the logistic regression equation (Table 3 and Additional file 1):

**Table 3:** Binary logistic regression between Metastasis and Ca-125 logarithm

	B	S.E.	Wald	df	Sig.	Exp(B)	95% C.I.for EXP(B)	
							Lower	Upper
Step 2^a^								
Age	0.053	.015	11.847	1	0.001	1.054	1.023	1.087
LOG CA-125	1.078	.185	34.010	1	< 0.0001	2.940	2.046	4.223
Constant	-8.163	1.415	33.295	1	< 0.0001	0.000		

^a^Variable(s) entered on step 1: Age, LOG CA.125

F = (Age*0.053) + [(logarithm of Ca-125) *1.078] – 8.163.

Were excluded non-significant variables named: “Pregnancies number” and biological status “Menopause” (Table 4). An internal validation was performed with the bootstrap method (Table 5).

**Table 4:** Binary logistic regression between Metastasis and Ca-125 logarithm, Age, Pregnancies number, Menopause

	B	S.E.	Wald	df	Sig.	Exp(B)	95% C.I. for EXP(B)	
							Lower	Upper
Step 1^a^								
Age	0.096	0.028	11.656	1	0.001	1.101	1.042	1.163
Pregnancies number	−0.098	0.083	1.406	1	0.236	.907	.771	1.066
LOG CA-125	1.034	0.185	31.236	1	< 0.0001	2.812	1.957	4.040
Premenopause/Menopause [1]	1.240	0.736	2.843	1	0.092	3.457	.818	14.619
Constant	−10.27	1.975	27.045	1	< 0.0001	.000		

^a^Variable(s) entered on step 1: Age, Pregnancies Number, LOG_ca125 (logarithm), State of Premenopause

**Table 5:** Bootstrap for Variables in the Equation

	B	Bootstrap^a^				
		Bias	Std. Error	Sig. (2-tailed)	95% Confidence Interval	
					Lower	Upper
Step 1						
LOG CA-125	1.078	0.049	0.215	0.001	0.781	1.639
Age	0.053	0.002	0.018	0.002	0.024	0.095
Constant	−8.163	−0.364	1.777	0.001	−12.597	−5.754

^a^Bootstrap results are based on 1000 bootstrap samples

### Diagnostic test of the metastasis prediction equation

**Fig. 1:**
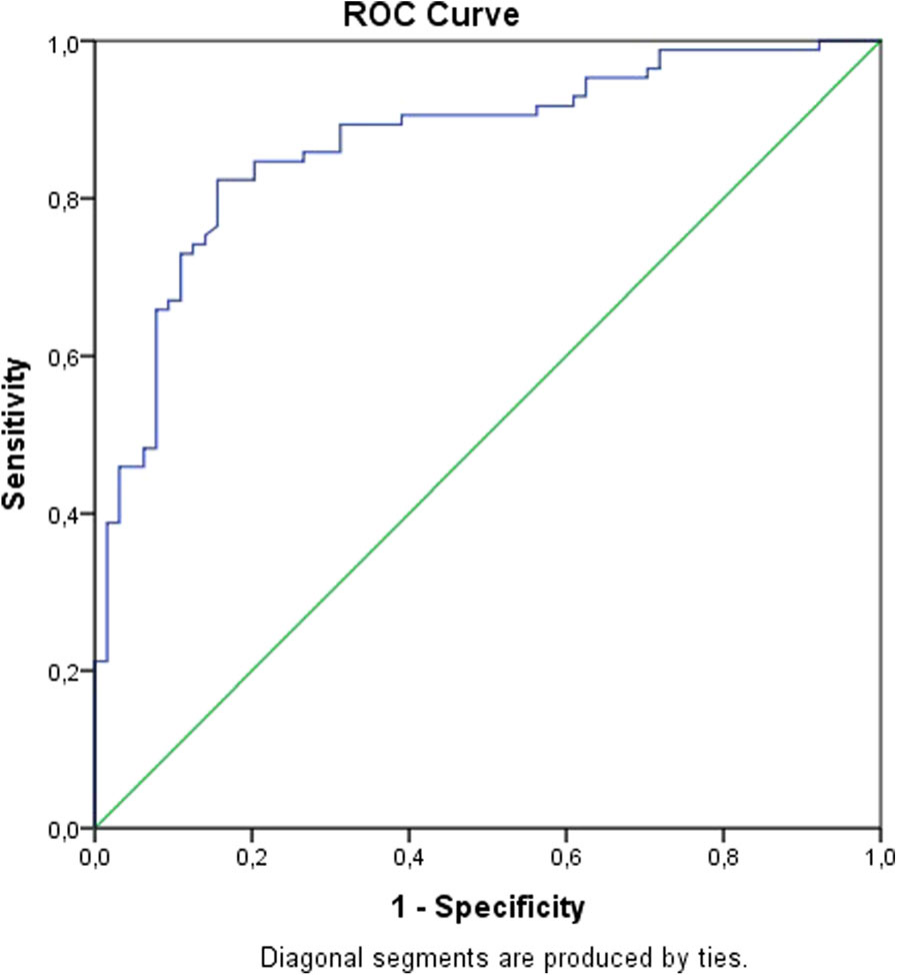
ROC curve between Metastasis and its prediction with an equation based on Age and Ca-125 Logarithm

A 2 × 2 contingency table was constructed. The data of groups 2 and 1 were placed in columns and the metastasis prediction equation in rows (Presence and Absence). Table values were (a) 70 cases (b) 13 cases, (c) 15 cases and (d) 51 cases. This table reported a sensitivity of 82.4%, and a specificity of 79.7%. The positive predictive value of 84.3% and the negative predictive value of 77.2%. The positive likelihood ratio was 4.059 and the negative likelihood ratio was 0.22. The ROC curve reported an Area under the curve of 0.870 (95% CI 0.812–0.928) with a standard error of 0.03 and *P* < 0.0001 (Fig. 1).

## Discussion

The main finding of the present study was that high levels of Ca-125 are related to the presence of metastases in this group of patients diagnosed with Ovarian Cancer. The Odds Ratio of this relationship is 2.940 (CI 95% 2.046–4.223) *P* < 0.001 (Table 3), the logistic regression equation was statistically significant and establishes the serum level of Ca-125 as a statistically significant predictor of the presence of metastasis. The equation had a positive predictive value of 84.3% with a positive likelihood ratio of 4.059. The ROC Curve reported an Area under the curve of 0.87 which was statistically significant. The cut-off point of the Ca-125 value to predict the presence of metastases in this study group was 240 u / ml. In the equation, non-prognostic variables were excluded, such as the fact of dying, and spurious variables such as family history and origin were excluded. The significance of finding an association between the stage of metastasis (State II and III of the FIGO classification) and the serum levels of Ca-125 allows us to have a practical and applicable equation in daily clinical practice. It has been established that the serum levels of Ca-125 pretreatment are increased, showing a positivity of 55% in the serous types, and between 42 to 77% in stage III [4] and stage IV (15%), being these mostly serous epithelial type [12]. In the present study, serous epithelial cancer was predominant (81.2%), and most of it was in stage III, which coincides with FIGO statistics [13] due to late diagnosis [14]. In the study it was found that the stage with the highest elevation of the marker was stage III (36.4%), this may be due to the fact that in advanced stages there is metastasis, while in the histological type it was the serous (85.27%), significant statistical relationship observed in other studies [14] however in no previous work an equation has been established to predict metastasis as in the present study. It has been shown that the association between Metastasis in patients with ovarian cancer and serum Ca-125 levels fits into the causality, since the tumor burden is intruded. The relationship is less associated with stages in which the load or tumor mass has been decreased in therapeutic form and may be an alternative explanation to the reason for decreasing the association with advanced stages already operated therapeutically. In daily practice, the equation helps to quickly predict the state of metastasis of patients and finally the mortality since the group with metastasis had higher mortality than the group without metastasis. This study does not include other tumor markers used in ovarian cancer. Future research should prospectively address the prediction equation to predict metastasis and compare it with a gold standard clinical.

## Conclusions

There is a relationship between the clinical stage of metastasis and serum Ca-125 levels in this group of patients with ovarian cancer.

## Additional files

Additional file 1: Excel sheet for calculating the probability of ovarian cancer metastasis. (XLSX 9 kb)

Additional file 2: Database in Excel format. (XLSX 19 kb)

## Abbreviations

CA-125: Cancer antigen 125
FIGO: International Federation of Gynecology and Obstetrics
